# Educators' Views on Using Humanoid Robots With Autistic Learners in Special Education Settings in England

**DOI:** 10.3389/frobt.2019.00107

**Published:** 2019-11-01

**Authors:** Alyssa M. Alcorn, Eloise Ainger, Vicky Charisi, Stefania Mantinioti, Sunčica Petrović, Bob R. Schadenberg, Teresa Tavassoli, Elizabeth Pellicano

**Affiliations:** ^1^Centre for Research in Autism and Education, UCL Institute of Education, University College London, London, United Kingdom; ^2^Joint Research Centre, European Commission, Brussels, Belgium; ^3^Serbian Society of Autism, Belgrade, Serbia; ^4^Department of Human Media Interaction, University of Twente, Enschede, Netherlands; ^5^Department of Psychology and Clinical Language Sciences, University of Reading, Reading, United Kingdom; ^6^Department of Educational Studies, Macquarie University, Sydney, NSW, Australia

**Keywords:** education, special education, schools, teachers, autism, children, humanoid robots, social robots

## Abstract

Researchers, industry, and practitioners are increasingly interested in the potential of social robots in education for learners on the autism spectrum. In this study, we conducted semi-structured interviews and focus groups with educators in England to gain their perspectives on the potential use of humanoid robots with autistic pupils, eliciting ideas, and specific examples of potential use. Understanding educator views is essential, because they are key decision-makers for the adoption of robots and would directly facilitate future use with pupils. Educators were provided with several example images (e.g., NAO, KASPAR, Milo), but did not directly interact with robots or receive information on current technical capabilities. The goal was for educators to respond to the general concept of humanoid robots as an educational tool, rather than to focus on the existing uses or behaviour of a particular robot. Thirty-one autism education staff participated, representing a range of special education settings and age groups as well as multiple professional roles (e.g., teachers, teaching assistants, speech, and language therapists). Thematic analysis of the interview transcripts identified four themes: Engagingness of robots, Predictability and consistency, Roles of robots in autism education, and Need for children to interact with people, not robots. Although almost all interviewees were receptive toward using humanoid robots in the classroom, they were not uncritically approving. Rather, they perceived future robot use as likely posing a series of complex cost-benefit trade-offs over time. For example, they felt that a highly motivating, predictable social robot might increase children's readiness to learn in the classroom, but it could also prevent children from engaging fully with other people or activities. Educator views also assumed that skills learned with a robot would generalise, and that robots' predictability is beneficial for autistic children—claims that need further supporting evidence. These interview results offer many points of guidance to the HRI research community about how humanoid robots could meet the specific needs of autistic learners, as well as identifying issues that will need to be resolved for robots to be both acceptable and successfully deployed in special education contexts.

## Introduction

Robotic systems targeted toward people on the autism spectrum, especially children, are a growing subfield of social robotics and human-robot interaction (HRI) research. Autism is a lifelong neurodevelopmental condition or spectrum of related conditions that affects the way a person interacts with others and experiences the world around them (American Psychiatric Association, 2013). Many autistic^1^ individuals also have additional difficulties with spoken language and/or intellectual disability, as well as co-occurring mental health problems, especially anxiety, and attentional difficulties—all of which can involve complex, long-term support needs. In England, ~120,000 children are documented as having autism as their primary form of special educational need and disability [SEND; (Department for Education, 2018)]. Of these, 28% percent of autistic children are educated in special schools and represent over a quarter of the total special school population. The children attending these schools often have complex needs, including an additional intellectual disability and/or limited-to-no spoken communication, and often require much higher levels of support from specialist teaching and allied-health staff than regular, mainstream schools can typically provide. These particular children are frequently overlooked by researchers (Tager-Flusberg and Kasari, 2013) but, along with the specialist staff that support them, represent two sizeable populations of potential robot users in England—and were thus the focus of the current investigation.

Autistic children are thought to be especially interested in and motivated by robots, potentially related to the fact that they are interactive—but programmed and ultimately rule-based—devices. Indeed, robot-based programmes are often cited to be potentially beneficial for this group in particular because they offer the possibility of fairly predictable and consistent interactions (e.g., Dautenhahn, 1999; Dautenhahn and Werry, 2004; Duquette et al., 2008; Rudovic et al., 2017; Straten et al., 2018). These are precisely the sort of interactions that autistic people are often said to favour (Pellicano and Burr, 2012; Lawson et al., 2014). The extant HRI literature suggests that autistic children may be highly engaged during robot interactions (Robins and Dautenhahn, 2006; Straten et al., 2018), and show spontaneous joint attention and other social behaviours that are often challenging for this group (Anzalone et al., 2014; Warren et al., 2015). Yet, existing research on social robotics for autism often constitutes proof-of-concept studies with small samples (*n* < 10), single rather than repeated robot-child interactions, and incomplete information about the autistic participants, making it more difficult to understand the potential applicability of the work as education or therapy [see reviews by (Diehl et al., 2012; Scassellati et al., 2012; Begum et al., 2016), for discussion].

Existing autism and HRI studies have predominantly studied children interacting with robots in lab-based settings (e.g., Salvador et al., 2015; Yun et al., 2016) or closely controlled, researcher-designed procedures that effectively re-create labs in schools (e.g., Kozima et al., 2007; Robins et al., 2012). Although there is much to be learned from studies in controlled lab-like settings, moving robots from the lab into the classroom (or “the wild”), where teachers apply the teaching programme unsupervised, is no straightforward task (Diehl et al., 2012; Huijnen et al., 2016). Embedding robots into existing autism contexts and pedagogical practices requires in-depth understanding of *specific* contexts and practices, and of the adult users who will support robot-based programmes. Understanding the views of these adults is therefore essential, as they are key decision-makers for the adoption of new technologies, and would be the ones to directly facilitate any future use of robots.

Several studies have sought teachers and professionals' views to explore implementing robots within regular educational settings (Fridin and Belokopytov, 2014; Kennedy et al., 2016; Serholt et al., 2017; Cheng et al., 2018) but only a handful have done so within special education settings. Diep et al. (2015) interviewed six teachers from a Canadian school for children with multiple and complex needs about their perceptions of social robots, in relation to an anticipatory governance framework (Guston, 2014). Although their results make some reference to autistic learners, they do not primarily focus on this group. In a larger study, Hughes-Roberts and Brown (2015) conducted interviews and focus groups with 20 teachers in special (though not autism-specific) education settings in the UK, incorporating a demonstration of a humanoid robot, NAO. Teachers stressed sustained engagement as a key indicator of success for many of their SEND pupils, and thus considered facilitating engagement as a key robot requirement. They highlighted three teacher-proposed robot activities, which included adults facilitating one or more children's game-like interactions. Perceived barriers to adoption focused on technical factors, describing the need for simple, fast, versatile, and usable robot controls. The only other limiting factor mentioned was the potential for robots to distract students from learning—at least while the robots were new. It was unclear, however, whether these educators considered, overall, robots to be relevant, appropriate, and feasible for their SEND settings and learners—and, most relevant to the current study, whether they might be especially useful for *autistic* learners.

Huijnen et al. (2017) took a related approach, combining focus groups, and co-creation sessions with autism stakeholders and professionals (including teachers and other school-based roles, all in the Netherlands) to develop 10 specific “intervention templates” for the humanoid robot, KASPAR. These included clear statements of goals, and explicitly mapped out the planned roles and “flow” of an interaction between a child, robot, and professional. This group discussed the role, requirements, and potential impact of the *adult* robot user in far more detail than any other study, ultimately “expect[ing] that the person operating KASPAR is a huge determiner of the success of the interaction and thereby of the intervention” (p. 3085). They also discussed characteristics or subgroups of autistic learners in relation to the suitability of robot use and, in a related paper, identified the potential educational roles that KASPAR could play, including those of a trainer, prompter, or mediator (Huijnen et al., 2019).

The findings from Hughes-Roberts and Brown (2015) and Huijnen et al. (2016, 2017) suggest that many educators seem to be broadly receptive—albeit cautious—toward at least some purposes of robots in autism or special education [though see (Diep et al., 2015), for more negative or mixed sentiments]. Educator interviews provide a valuable starting point for understanding whether and how robots might be integrated into existing educational practices, and might transition into being teacher- (not researcher-)managed tools. Yet, these studies only give a partial picture of the information researchers need to know to work toward robot deployment with autistic learners within special education settings. This is for three key reasons. First, these learners' specific needs and the strategies used to support them can be very distinct from those educated within mainstream settings (Eaves and Ho, 1997). Greater knowledge is needed about the utility of robot-based programmes for these particular children in their own specific, specialist contexts. Second, these and other existing studies have frequently asked educators to answer questions or discuss ideas in relation to demonstrations of existing robots (e.g., Hughes-Roberts and Brown, 2015; Coeckelbergh et al., 2016; Huijnen et al., 2016; as in Cheng et al., 2018). This approach can be useful if the goal is to generate or assess applications for those specific robots, but it is necessarily limiting with respect to discussing perceptions and applications of robots as a *category* of tools, or for generating novel use cases, as it primes participants to think of *that specific* robot when developing their ideas. Third, much existing research has either used surveys and questionnaires (e.g., Coeckelbergh et al., 2016; Kennedy et al., 2016; Cheng et al., 2018) to ask educators to *respond to topics and ideas that have been pre-identified by researchers*, or, have effectively leveraged educators' expertise for solving particular design or pedagogical problems (e.g., Huijnen et al., 2016, 2017). Educators' priorities and ideas about robotics might be different than those of researchers, but existing work seems to have given limited opportunities to explore these issues.

The current study is part of the European Union funded DE-ENIGMA project (de-enigma.eu), in which teams with technical and autism education expertise are collaborating to explore the potential of humanoid robots as tools in autism education, particularly with respect to teaching social and emotional skills, and to develop real-time multimodal processing of autistic children's behaviour. One strand of the project sought to better understand current specialist autism education settings in England, i.e., the target users and context of use for DE-ENIGMA outputs. This paper reports Part B of a two-part interview study with autism educators. We focused on educators, rather than a wider range of autism stakeholders, because DE-ENIGMA's focus has been specifically on schools. Part A (reported in Ainger et al., Manuscript in Preparation) investigated autism educators' current goals and pedagogical practices. Part B, reported here, discussed the potential future use of robots.

Our goal in Part B was to elicit educators' views and perspectives on the potential use of humanoid robots with autistic learners in special schools, to better understand the factors perceived to be important for deploying robots in these settings. We also focused on understanding educators' perceptions and suggested applications of humanoid robots as tools for teaching social and emotional skills, due to the focus on this topic within the DE-ENIGMA project. Unlike some previous studies that have asked educators to respond to ideas and topics pre-identified by researchers (e.g., in surveys and questionnaires), we used a semi-structured interview schedule, with researchers exploring participants' ideas in detail, following from fairly open questions.

## Materials and Methods

### Participants and Educational Settings

Thirty-one educators (female: *n* = 25) took part in individual semi-structured interviews or small focus groups, between December 2016 and January 2018. These educators were recruited via convenience sampling through existing community and personal contacts. All of our participants worked in specialist settings in England: 26 in special schools (*n* = 7, autism-specific; *n* = 18, general SEND), five in autism resource bases attached to a mainstream school, and one working across multiple SEND settings.

Autistic children educated in special schools in England usually have a high degree of adult interaction and support throughout the school day. In special schools, classes are small (often 5–10 children), with a highly trained teacher and a team of teaching assistants, who often have less specialist training. There is further input from specialist allied health professionals, including speech and language therapists and occupational therapists. Consistent with this context, our participants reported working with learners on the autism spectrum in a variety of educational roles, including as a primary (*n* = 12) or secondary (*n* = 5) teacher, teachers working across multiple ages and/or school settings (*n* = 2), a teaching assistant (*n* = 2), a headteacher or deputy headteacher (*n* = 3), a speech and language therapist (*n* = 3), or an occupational therapist (*n* = 2). Many participants indicated more than one autism-related role and had worked with multiple age groups over time, from Early Years education (<5 years), up to age 18–19 years. They varied widely in their level of experience, ranging from <1 to 18 years' experience in their current education setting (*M* = 4.7 years, *SD* = 4.1) (see Supplementary Table 1 for participant details).

### Procedure

Fourteen participants (female: *n* = 11) completed individual, semi-structured interviews in a quiet room at the university or school, and 17 participants took part in one of three focus groups (female: *n* = 14) in participating schools (two groups contained six participants, one contained five), facilitated by a researcher (see Supplementary Table 1). Part A of the interview study (Ainger et al., Manuscript in Preparation) focused on current educational contexts and practices, including participants' aspirations for their autistic students, their views on how social and emotional skills are currently taught within classrooms, curricula and supports used in their setting, and uses of technology (see Supplementary Table 2). To introduce the discussion of humanoid robots in Part B, the focus of the current study, participants viewed six example images of existing robots (Milo, KASPAR, NAO, Flobi, PARLO, and Pepper). They were not given any further information about these particular robots, their current capabilities, or examples of use and were encouraged not to be concerned about issues of technical feasibility. Instead, they were asked to consider the potential uses of humanoid robots for autistic children's learning, including potential benefits and concerns (see Supplementary Table 2).

While the interview did not explicitly ask about respondents' prior experience with or knowledge of robotics, almost all educators stated that they had no prior experience or knowledge of robots. The exceptions were one educator working with older students, who reported using commercially available Bee-Bot® robots to teach science and programming, and some educators who had seen previous demonstrations of a humanoid robot (Milo) in connection with the DE-ENIGMA project.

The protocol was approved by UCL Institute of Education Research Ethics Committee (REC857). All participants gave written informed consent to the interviews, including audio recording, in accordance with the Declaration of Helsinki. The total duration of the individual semi-structured interviews lasted 30–54 min (*M* = 40 min) and focus groups lasted 52–78 min (*M* = 62 min). The robotics-focused questions (Part B) lasted 5–12 min in individual interviews, equating to 14–31% of the total time (*M* = 8.5 min, 20%), and the robot section of focus groups lasted 15–18 min, or 24–35% of total discussion time (*M* = 17 min, 29%).

### Thematic Analysis

Audio-recordings were transcribed verbatim. The robot interview data were analysed using thematic analysis (Braun and Clarke, 2006), which included familiarisation of the data; generating of initial codes; generating themes, reviewing, defining and naming themes; and compiling this report. We adopted an inductive approach (i.e., without integrating the themes within any pre-existing coding scheme or preconceptions of the researchers) within an essentialist framework (to report the experiences, meanings, and reality of the participants). Two authors (AA and EP) independently familiarised themselves with the data and liaised several times to review the themes and subthemes, focusing on semantic features of the data, resolving discrepancies and deciding the final definitions of themes and subthemes. Analysis was thus iterative and reflexive in nature. Participants' responses to Part A of the interviews, on current educational goals and practices, were analysed and reported separately (Ainger et al., Manuscript in Preparation).

## Results

**Figure 1:**
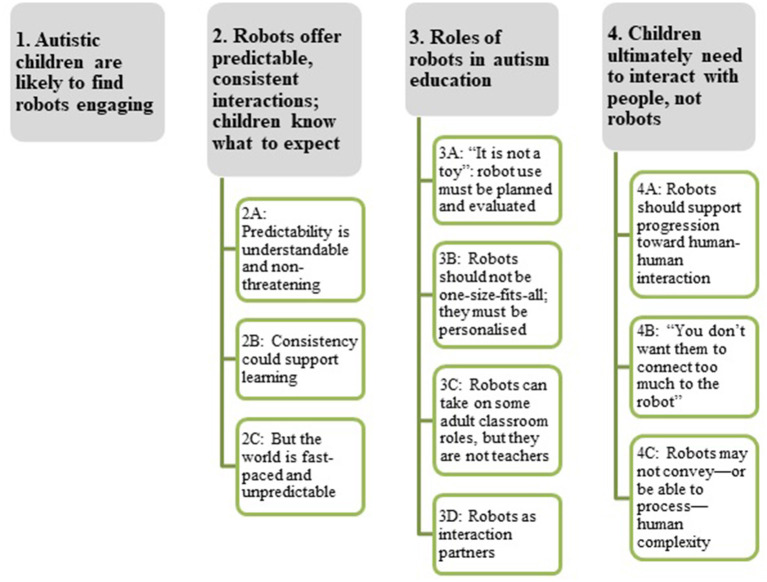
Summary of themes and subthemes.

We identified four themes in educators' interviews (see Figure 1 for summary of themes and subthemes). Throughout, educator quotes are attributed via participant ID numbers.

### Theme 1: Autistic Children Are Likely to Find Robots Engaging

Participants stressed the importance of engagement and motivation for learning, and anticipated that the autistic learners in their settings would be “so interested” in and motivated by humanoid robots, potentially more motivated than when interacting with adult educators, or non-technical activities. One explained: “I think if the robot's doing it [modelling behaviours], it's more captivating than just us as a person. This is a toy that plays back essentially, it's engaging” [101]. Educators also felt that this engagement could have a positive impact on their readiness to learn: “They would be really happy to work with it for longer periods of time, much longer than usual because, let's be honest, a piece of paper and a worksheet, it's not as exciting as a humanoid robot can be” [011].

Participants reported that, for some children, the attraction of a humanoid robot might be sufficient to encourage them to engage in otherwise challenging social interactions: “engagement is a big key to the social barriers that children may face, and if they're able to engage and experience some of those interactive activities, which they avoid at all cost in other settings… I really think [a robot] could support the social skills” [004]. Yet, robot attractiveness and engagement were not perceived as wholly positive. Respondents often discussed this characteristic alongside potential drawbacks, including concerns “about the extent we're going to use the robots… when we're talking about autistic children, we need to be very careful with something [not] to become an obsession” [011]. Another educator commented: “particularly with the younger ones with autism, we're trying to make them think that people are amazing… so all the teachers in the sessions try to become the most exciting thing in the room” [105]. For some children, educators further felt that access to a highly attractive robot could conflict with overarching educational goals to help autistic learners attend to and understand other people (see also Theme 4B).

### Theme 2: Robots Offer Predictable, Consistent Interactions; Children Know What to Expect

Educators in this study expected humanoid robots to be “consistent” and “obviously predictable” compared to people, who “behave in all sorts of different manners and ways” [015]. One educator summed it up:

“Robots, unlike humans, they will always be the same. Their tone of voice will always be the same, their inflection will always be the same, the body language is always the same. They're very predictable, like if you say a certain thing, it will say a certain thing back to you. So I think with kids with autism, they love that kind of thing, predictability” [014].

Overwhelmingly, they saw predictability as a potential benefit for their students but, as in Theme 1, they frequently discussed this benefit alongside less-positive implications.

#### Subtheme 2A: Predictability Is Understandable and Non-threatening

Educators emphasised autistic children's difficulty in making sense of other people's often-unpredictable behaviour: “this is a struggle, they cannot predict people but a robot is quite predictable with its reactions” [017]. Robots were perceived to be “easier” for children because “they know what to expect” [010] and could help them to “predict what might happen” [015].

Educators often talked about the importance of their students feeling safe and secure, and thought that “a robot like that would be something safe for my students, safe to interact, safe to communicate… they wouldn't feel threatened” [008]. One specific, anticipated benefit of a robot's predictability was that children might feel more at ease interacting with robots, relative to how they feel in other school activities or human interactions: “These children might respond to the robots better than the way they respond to other people because they might predict their reaction. So, for example, if they know that when they say ‘happy' he smiles, it could be less scary for them” [002]. Some educators also felt that this benefit could have a positive impact on their learning:

“Many of my students won't push themselves harder because they are afraid of making a mistake. Maybe if a robot like that would exist in my classroom, they wouldn't feel so intimidated or threatened from the teacher's authority and they would be able to try different things and that would help them progress and develop in different aspects” [008].

#### Subtheme 2B: Consistency Could Support Learning

Respondents also highlighted the possible benefits of a robot's consistency or “sameness,” particularly in its visual appearance and manner. One educator remarked:

“We do have different people coming in as supply teachers or supply TAs [teaching assistants] for the day and, if some of the students do not like the way someone is dressed or smells or talks to them, they won't communicate with them. But a robot like that will have the same specific characteristics every single day and that's something that would be very useful for my students. They will know that this robot would look exactly the same every day and they will be able to build a trust with the robot and communicate more” [008].

Another respondent suggested using the robot for helping to focus their attention on academic learning due to their unchanging manner and appearance: “[autistic students] can only concentrate to the words that the robot says. When we [staff] used to teach them, they could concentrate on everything else on us, like the way we move our hands, the way that our hair is today. So I think a robot could actually attract their interest on a specific thing that we want them to learn” [005].

Educators also used “consistency” to refer to a concept sometimes described in the autism-robotics literature as *repeatability:* a robot could repeat usually-variable social behaviour (e.g., a facial expression) over and over, helping autistic children to begin identifying patterns and associating meanings with the behaviour.

“The challenges of face-to-face and eye contact and response to facial expression and understanding somebody's facial expressions are so inconsistent that, with a robot, [autistic children] can start to learn what those consistencies are and it becomes much easier for them to respond to them, rather than a human facial expression, which could mean all kinds of things. I think with a robot they learn very quickly… [they] may start to associate meaning with some of those facial expressions and recognise those in others and maybe seek some of those communicative responses” [004].

Educators also felt that robotic consistency might be particularly advantageous if applied to classroom interventions that *require* consistency and rule following, such as the Picture Exchange Communication System (PECS; Bondy and Frost, 1994), a widely-used alternative/augmentative communication system. Indeed, they felt that a robot might deliver such an intervention with *more* fidelity than a human teacher:

“The PECS system is very definite and it's very, very rule based, but as humans, there's distractions and that means the delivery of this rule-based training we often get wrong. Robots would do it consistently so that a child, an autistic child working with a robot that's programmed to deliver training only in that specific way following that specific algorithm, [the child is] going to respond much better because they're getting a consistent response. So I think you'd have better outcomes if robots are teaching autistic kids certain protocols” [013].

#### Subtheme 2C: But the World Is Fast-Paced and Unpredictable

Educators repeatedly highlighted that, unlike the expected behaviour of robots, both humans and life are *un*predictable, and that one key educational goal was to support children in learning to deal with this uncertainty. Educators were concerned that predictable and consistent robots would potentially hinder children's progress in this regard: “[technology], largely speaking, you know, does what they want it to do. What we want them to understand is that the world is unpredictable and the world has huge variety in it, and we want them to be able to respond quite flexibly to things, as well as follow somebody else's agenda” [201].

Educators noted that, while a robot might not “mind how long it takes for a child to do anything…it could be really deskilling for the child because you don't have all the time in the world with a robot waiting for you when you're an adult, like you do have to just go on the bus and swipe your [bus pass], you do just have to transition” [103]. Transitioning between activities and/or settings can be an area of particular difficulty for autistic children.

Our participants also felt that, while children might learn more easily or feel more comfortable with a highly predictable robot than when learning with a person, that type of learning could be counter-productive in the long run because it does not support skill generalisation: “I don't know, maybe it's going to be too predictable for them, and then how will they generalise when they actually have to interact with actual people. So maybe by teaching them this predictability, it's not that easy to help them generalise it” [010]. Some educators reported that a robot could provide a “good base” for teaching simple social skills but warned, “if our goal is to teach kids social skills and interaction and how to interact into the world and the community, then that's not through robots because at the end of the day, our community and our real world are not made of robots. So it's very important that we phase out a bit and then have more human contact” [014].

### Theme 3: Roles of Robots in Autism Education

Educators' examples of how robots could potentially be used varied widely depending on their settings and the profile of their learners. Nevertheless, there were several key commonalities across the interviews.

#### Subtheme 3A: “It Is Not a Toy”: Robot Use Must Be Planned and Evaluated

Educators agreed that robots are “not a toy.” Rather, any use of social robots in their settings would need to be planned by teachers, “really thinking carefully, ‘How do I use it? Is it appropriate?”' because a robot “might not be appropriate for every single child” [203]. Some framed the need for planning in relation to their past experiences with iPads in class. Like robots, iPads were perceived to be attractive, flexible technologies but, according to educators, were often introduced without clear goals, creating knock-on problems in which autistic learners might “see an iPad or a technological device as something that is mainly a toy. They can develop some obsessive behaviours or they will be repeatedly asking for an iPad without completing the work” [008]. One interviewee neatly summarised: “I don't think [robots] should end up being used like iPads, just for fun and just as a toy. I think they, when you use them, should have a very clear target for why you're using it and for a very clear amount of time and with a purpose” [011].

Indeed, educators emphasised that educational planning would therefore need to consider whether the robot was “appropriate for every single child…really just thinking carefully, like everything we do here, ‘Oh is that child ready?' and to really teach something specific, not thinking just putting them in the same room with the robot and then leave and think they'll know everything” [204]. Another focus group agreed: “It wouldn't have to be like, ‘okay, now we're learning the social skills next time the robot is coming up' but I would look at each child and see like, ‘okay, how am I going to use it with that learner' and then find a time and a setting that feels appropriate” [302].

One respondent further suggested that planning to introduce robots or any new tool must incorporate *evaluation*, perhaps especially if teachers have high expectations and perceive the new tool as a “[scheme] that they believe will work and will fix everything.” She noted,

“We say, ‘oh yes, try that, that might work,' and there's nobody assessing as to whether or not it is working. We need a baseline check to start with and then we need to check whether or not it's worked at the end of the intervention. Interventions are incredibly expensive so therefore you have got to have the mindset that you're going to look to see whether that intervention has worked” [007].

#### Subtheme 3B: Robots Should Not Be One-Size-Fits-All; They Must Be Personalised

In autism education, “personalisation” is a fundamental task in which educators choose, adapt (and often, invent) tools, and strategies “that are catered to that child” [301]. The educators we spoke to clearly expected that the same type of fine-grained child-level personalisation would be necessary and “programmable” with robots, in addition to choices about the types of learning activities different students may do: “[robots have] got to be based on the likes and dislikes of the child… the adjustments would be, you know, that [the robots are] programmed to do a variety of different things” [013].

“If I have a very verbal student who just needs to practise reciprocal conversation or needs to practise its tone of voice or practise identification of feelings and expressions, then I'd program the robot for that. But then if I have only that one robot but then I want to use it with a different kid, who's non-verbal, doesn't like interacting with people at all, then I would have the robot programmed to not say anything, to not maybe do any sudden movements. I would program it depending on what level the student is or what social skill I want to work on” [014].

One respondent agreed wholeheartedly with the importance of technology personalisation, but questioned how well teacher-implemented robot personalisation would work in practice, based on their current experiences with a dyslexia-focused app, Wordshark (https://www.wordshark.co.uk/). She described how this program “can be tailor made to fit the particular child and quite often teachers don't use that tailor-made bit. They just think, ‘oh yeah, Wordshark, Wordshark is supposedly very good so let's use it,' and they're not using it in the way that the manufacturers intended” [007]. She also pointed out that these issues around personalisation and correct use can be exacerbated by school-level decisions around technology and training, in which institutions “invest in one particular member of staff, ‘here you are, you're the expert in this' and then either that trend is not cascaded down, or that person then leaves and the technology is left behind and nobody really knows how to use it.”

#### Subtheme 3C: Robots Can Take on Some Adult Classroom Roles, but They Are Not Teachers

In their discussions, interviewees' suggested robot roles reflect the types of routine support that staff members offer autistic children throughout the day, including “to guide them, to give them ideas, and maybe even to prompt them or to praise them” [008], “especially the higher ability ones, who when I leave them to work independently, they lose track of what they're doing” [009]. Others also felt that educators “could use it as a tool as a part of the group, so the robot could almost form part of the group or it could be used as, it might lead the session or the group” [302].

Yet, while the interviewees suggested that robots could usefully offer some types of support and facilitation currently provided by various adult staff, other discussions made clear that the robot was not seen as a potential *teacher*. Educators emphasised that “the adult always needs to be in control with what's happening” [303], especially with regard to planning and goal-setting. Where some respondents indicated that robots could be adaptively responding to children, these comments were always made within the context of supporting educational or social goals already identified by teachers. There was no discussion of future humanoid robots “assessing” or identifying children's needs.

Beyond issues of planning and control, respondents pointed out that special education teachers are trained in a distinct set of skills and strategies that they need to support their learners. Educators were concerned that reliance on a robot may both deprive autistic learners of the benefit of those skills, but also (over time) detract from staff members' ability to exercise those skills. One focus group participant explained:

“Part of our skills we have as special needs educators is that we're able to empathise, and use lots of creative strategies, to the point where you understand why someone finds it challenging to transition and hopefully don't find it so frustrating anymore. I think it's important to swap around as a team as well, not just leave it to a robot” [102].

This “professional deskilling” concern was shared. Another educator noted their own lack of robot experience and training, explaining that their “main concern is whether I would be able to use it appropriately and I wouldn't lose other aspects of my teaching. For example, I wouldn't want to rely too much on the robot to communicate with my students or to help my students access the knowledge” [008].

#### Subtheme 3D: Robots as Interaction Partners

Even before being explicitly asked about possible applications of humanoid robots to social and emotional skills teaching, respondents spontaneously suggested social applications and roles for the robot. As they had explained earlier in the interviews (Ainger et al., Manuscript in Preparation), “the most important goal is to help them progress with their social skills” [010]. Teachers believed that attractive robots might act as *social partners*, motivating children to work on inherently challenging social and communication skills that are already targeted in existing class activities, such as turn-taking in activities (“You're waiting for the robot to finish talking and then it's your turn to talk, so it's like turn taking, you know, how to have a conversation with somebody” [011]) and conversations (“like having kids just learn general conversations like teach them to say, ‘hi, my name is A, what's your name? How are you feeling today?' Like just have them practise conversations, have them practise answering questions but also having the kids practise coming up with questions themselves” [014]).

Educators specifically highlighted the role a robot could play in understanding how children's own behaviour affects others—one of “the biggest thing[s] for our learners” [302]. Another interviewee concurred that some autistic learners “cannot see how the way that they're behaving affects other people. So this would be a nice thing to use the robots for… [learners] could perhaps see how their behaviour was affecting somebody else” [007]. Other respondents gave specific examples of how they might work on this concept, using the robot, including “a programme for how to make the robot happy today… The programme might ask for some steps that the child has to do like feeding or giving water or going for a walk or holding hands or playing a game, whatever makes a robot happy” [017]. Another offered:

“I just think of like a robot crying and then having like props of tissues or whatever, you know, and then making my children try to calm him down… care for the robot as well, you know, when he says that he's angry or he's got a cut in his wrist or something, I think they really could connect with that. I think that could be a great tool actually” [015].

Educators also suggested that understanding cause-and-effect with the robot could also be used to go beyond grasping event relationships, to “build that empathy and understanding [of] other people. So, the child is angry and might be pulling or shaking the robot or hitting the robot, that the robot might be able to respond to that in a way that it's communicating to the child how those actions are making him feel” [304].

Respondents were not universally approving of using the robot to teach social communication. One respondent was receptive to the idea of robots in general, saying “with the right software or the right purpose, it could be awesome,” but was emphatic that its uses should not include anything “related to emotions or behaviour management or any patronising sort of thing” and “nothing like engaging in social skills or emotional stuff” [016]. This same respondent had expressed particular concern about the robot's capacity to meaningfully render complex human behaviour, and to respond appropriately to autistic children.

### Theme 4: Children Ultimately Need to Interact With People, Not Robots

While they expressed interest and cautious optimism about the use of humanoid robots in autism education, interviewees were also very clear that robots were perceived to have potential and acceptability primarily as “stepping stones” to fostering human-human interaction.

#### Subtheme 4A: Robots Supporting Progression Toward Human-Human Interaction

Respondents either implicitly or explicitly indicated that working with a robot in a school context would be a transitory, middle phase between two different *types* of human-human interaction. Educators felt that they would first need to introduce the robot “in a familiar space, with trust and familiar adults that can say, it's okay” [301]. Many autistic children are highly anxious about all new people and activities, and staff suggested addressing this issue using existing educational strategies such as “a social story about it, [showing] pictures beforehand, [explaining] what's going to happen with the robot, when the robot will be coming” [301] (see Gray, 1994, on social stories). These steps, which can “build up almost the story of this robot, how it's coming here, and when it arrives then the pupils will probably be more—shall we say, prepared for its arrival” [101], are useful for any child's interaction with a robot, but especially so for autistic children, who require additional preparation to adapt to novel objects and events their environment.

Educators then described how children might work with the robot on skills or activities over time, again potentially supported by some degree of adult guidance: “that's one of our targets, especially in my class, is getting kids to talk to one another. So that could be almost the first step, rather than talking to an adult, you're talking to the robot” [305]. At a later point, children might transition away from work with the robot, applying those skills in interaction with peers, adults, or the community: “You can practice having conversations, you can have the robot opposite you and you can set certain rules and you can first practise with robots before you move on to adults” [011].

Respondents suggested that humanoid robots might be particularly successful at supporting social learning and later generalisation, because “the fact that it is human-like might help them to associate the robot with human behaviour.” Another explained with reference to the robot image examples provided in the interviews/focus groups: “I prefer the ones that look more like a human. Most importantly, it's going to be like it's a real boy, it's a real-life example. They would consider the rest like a toy but this [humanoid robot] might be actually an example” [010]. Other educators felt the opposite, that human-like robot appearance and behaviour could be confusing and create problems: “I think that will be my main concern, you know, how to explain to the child that this is only a robot, it doesn't have feelings, and it's different than mum and dad or friends and teachers” [015]. Another agreed that “we don't want them to start thinking this is a human, ‘this is my friend' or ‘It's the same as my peers”' [204]. Others thought children's understanding of robot-human differences would be dependent on their age and cognitive ability, and one respondent flatly dismissed these concerns, maintaining that to “someone who has autism, a robot is a robot, even if it looks like a person” [016].

#### Subtheme 4B: “You Don't Want Them to Connect Too Much to the Robot”

Educators expressed concern that children might have “too much” interaction with a humanoid robot, in various ways. Some perceived time spent interacting with the robot as directly detracting from time spent with people: “with my kids, you know, [my concern] would just be maybe about the amount of time they would be engaging with it and making sure that they're not always engaging with the robot and they're engaging with other children” [302].

Our participants were also worried about children's emotional investment in the robot. They felt certain that autistic learners could trust and emotionally connect with a robot—perhaps more so than with a person: “You don't want them to connect too much to the robot, that then it's almost like an imaginary friend, like that they rely so heavily on this robot that then they don't socialise” [303]. Another predicted: “they will become too dependent, they will prefer to be with the robot than be with mum or be with sibling and interact with friends. I would be just scared that they will get too attached. I would rather see my children interacting and playing with me or with each other than with the robot” [015].

Suggested applications where children would “build up” from robot interactions to human interactions were repeatedly positioned as a way to balance the potential benefits of supportive, reciprocal robot interactions with the risk of these overshadowing existing relationships. One participant summed this up:

“I feel that a robot will work more or less in the same way as our students. There would be a common ground to communicate and share feelings and emotions, a better way to express those emotions instead of interacting with an adult, or their peers. And I'm not saying necessarily to interact just with the robot because that would lose their communication part with, the other human beings in the classroom, with the adults or with their peers. But I think that would be the first step for them to start expressing their feelings and emotions and then it would be easier for them to involve other human beings in the classroom… [in] their everyday lives and showing their emotions and communicating their needs” [008].

#### Subtheme 4C: Robots May Not Convey—or Be Able to Process—Human Complexity

Educators repeatedly noted the complexity of human behaviour, and were concerned that humanoid robots' behaviour would lack nuance and variation, particularly for social communication: “You could teach a robot to do this and that but not everyone does it the same way. One person when they're angry might cross their arms but some people might tap their foot. So human behaviour is so erratic and unpredictable and everybody's behaviour for whatever emotion is different” [001]. Educators felt that this lack of variation would limit the robot's potential with regard to what it *could* teach: “With autistic kids, certainly they could mimic [the robots] but because they could mimic them, they would be in risk of learning one expression for one feeling and that's not right ‘cause the diversity of emotions is so wide and the way we adjust and the way we process emotions is so different” [016]. As with the mixed implications of robot predictability and consistency (Theme 2), educators felt that a robot that is programmed to—or is physically limited to—showing a social behaviour in only one way might potentially do autistic children a disservice by not preparing them to understand the true range of human behaviour. They also described how a real, two-way exchange of feeling would be missing: “Social interaction is emotional for both sides, so it's something more than you just get with the robot who is just there, he's predictable. Human relationships are much more complex than the robot I think can show” [104].

Other concerns focused on how the underlying technology would not be able to adequately cope with—and adapt to—the diversity and unpredictability of autistic learners' behaviour: “Even if our students are very structured and predictable, they can also be unpredictable and I don't know if a robot could be able to adjust to those things” [013]. Additionally, “I doubt that a robot could recognise the different ways a person with autism could express [the] same emotions. I think it would be hard to design a software for that” [016].

## Discussion

In this study, educators were provided with minimal information about what humanoid robots “are like” or their current or future uses to avoid biasing educators' reflections toward specific, existing examples. Educators were therefore free to project their own ideas of whether, and in what ways, future humanoid robots might contribute to autism education. This approach differs from some recent practitioner studies, where participants were introduced to specific robots, or were asked to solve specific problems (e.g., whether KASPAR could add value to a particular learning domain; Huijnen et al., 2016). Overall, the current respondents were open to discussing humanoid robots within autism education contexts. They expressed a willingness to find out more about them, or to try interacting with them for themselves to see what their capabilities might be. These respondents from autism education settings shared many basic perceptions of robots with both the mainstream, UK-based educators in Kennedy et al. (2016), including robots as having “simplistic interactions” and being “primarily seen as a scripted, reactive machine” (p. 5), and with the Canada-based special educators in Diep et al. (2015), who felt that robots might “[provide] structure and repetitiveness in a consistent fashion” (p. 2). Yet, the same qualities that our participants saw as potentially so promising for meeting the needs of autistic learners were perceived as *obstacles* to adoption by the Kennedy et al. (2016) mainstream sample (see also Serholt et al., 2017); an illustration that “educators,” “autistic children” and “schools” are not homogenous groups and will have different needs—which need to be fully understood to inform future robotics work.

Our respondents' openness to discussing future robot use did not equate to unqualified endorsement, however. Where educators predicted that robots could benefit their learners, these predictions were both conditional and carefully circumscribed: robots may be beneficial, *if* used in a certain way, and *if* certain measures are in place. These circumscriptions consistently position proposed future robot use within established educational goals and supports. Educator responses also revealed a shared prediction that any future robot use would pose a series of complex cost-benefit trade-offs: if a robot is appealing and motivating, it may become a liability if children engage with it to the exclusion of other interactions; a predictable robot could support short-term learning goals, but might then interfere with children's longer-term capabilities to cope with a mutable world. As part of their initial consideration of whether robots belong in autism education, teachers were already looking at the implication of robots across a child's school career, or their lifespan. Such predicted trade-offs must be addressed by carefully planning robot use, within existing practices and within individual learners' pre-existing goals (subtheme 3A). Autism specialists in Huijnen et al. (2017) made similar comments on the imperativeness of planning robot use, though did not discuss its longer-term implications and trade-offs as did the current participants. These perceived benefits and trade-offs have significant implications for the autism-robotics field, and will be discussed in turn below.

### Robots Are Novel, but Not Different From Existing Tools

Across all of the interview prompts, educators discussed humanoid robots in a remarkably similar way. Interviewees proposed robot uses that supported existing curricular goals, and volunteered a range of established educational strategies that could be applied to introduce robots and support their use. Suggested robot activities and roles built on existing classwork (e.g., practicing turn-taking in a small group) and staff roles. Respondents' emphases on cause-and-effect and turn-taking, plus the specification that adults must be present to support robot use, echo the teacher-proposed robot learning activities in Hughes-Roberts and Brown (2015) and indicate that social skills practice with robots has wider relevance for special education populations.

Humanoid robots are a novel technology to autism educators, and one for which they can propose possible applications. However, the current interviewees did not have an expectation of robots affording *completely new* educational goals, but rather, of robots representing a potentially powerful tool to pursue existing goals. Overall, humanoid robots were not perceived as being fundamentally *different* from current, widespread technologies, such as tablets. Autism specialists interviewed on their existing iPad use in King et al. (2017) described comparable patterns of use to those that our respondents envisioned for robots, “attempting to integrate tablets into the standard instructional methods that they were already using” (p. 9). To the current respondents, humanoid robots could be fully compatible with current autism education practices, *if* they can support key longer-term priorities (see Generalisation and Effectiveness: Challenges to Educational Robot Adoption?). This perceived instructional compatibility does not negate the desire for specialist training about robot use, and for that training to be distributed across school staff. Respondents in Huijnen et al. (2017) and King et al. (2017) made similar points about KASPAR and iPads respectively: they wanted training both on how to operate the devices and how to make the most of them pedagogically.

As with any educational tool, educators indicated that humanoid robots should be one component or phase of educational activity that is carefully planned to integrate into wider practices; participants in Huijnen et al. (2017) similarly stressed the need for integration. Lesson planning, introducing the robot, and—eventually—transitioning to human interaction were envisioned as being planned and managed by teachers. At least some teachers also seemed to envision taking responsibility for programming robots, or otherwise adapting them to individual learners (see Personalisation, Content, and Teachers-as-Programmers). Respondents' examples of potential robot use implied that some degree of autonomous behaviour would be acceptable and useful, such as robots being able to respond to children in an ongoing activity, to detect when children need prompting, or to offer praise. In Huijnen et al. (2016), participants suggested similar preferences for “semi-autonomous” robot operation with autistic learners with specific reference to the existing KASPAR platform. However, some current interviewees raised the concern that robotic technology may not be well-equipped to autonomously interpret and respond to autistic children's variable behaviour.

Even if robots do not demand new ways of working, interviewees still identified areas of desired improvement from existing practices around technology use in their schools. They clearly had mixed experiences with iPads in particular, as devices that could be *too* engaging, and specifically referenced them when emphasising the need for careful lesson planning around robot use. Once again, there is close alignment between these respondents' views and those reported in King et al. (2017), in which educators acknowledged “numerous challenges” of iPads such as “perseveration,” but yet retained “an overall optimism about tablet use. They were aware of the incredible motivation tablets provided for [autistic children] and realised their potential across several areas” (p. 8).

One area in which the current results *differed* from other teacher studies on robots or iPads was the degree of concern over children becoming too emotionally attached, or robots potentially detracting from children's peer, family, and staff relationships. This is more specific than concerns over the amount of use, and also seems distinct from concerns about technology isolating autistic children (e.g., King et al., 2017). This may be one area in which *humanoid* robots are perceived as special and facilitative of social relationships with autistic children in a way that other devices may not be. However, as with other robot characteristics, human-ness and social capacity were also perceived as pedagogically important (subthemes 3D, 4A). These concerns about overly close and important social relationships with robots are diametrically opposed to some of the Canadian special educators' opinions in Diep et al. (2015), where “face-to-face interaction was seen as an important task they felt the robot could not provide” and that robots “cannot perform the task of providing emotional comfort or communication” (p. 2). These divergent views may indicate both differences of opinion between groups of educators, but also views of robots shifting over time (data from Diep et al. were collected from six teachers in 2012) as technology becomes more sophisticated and is increasingly publicised.

### Generalisation and Effectiveness: Challenges to Educational Robot Adoption?

When asked to discuss potential applications of humanoid robots, educators consistently talked about them as a “stepping stone” to learning, between an introduction that is carefully managed by school staff and a supported transition away from the robot, toward applying new skills with human partners. Endorsing this basic three-stage pattern of robot use appeared to counteract some respondents' concerns about the possibility of children becoming overly reliant on robots, or interacting with them at the expense of classmates and families (subtheme 4B), and made them more ethically acceptable. The stepping stone pattern also relies on educators' special skills and knowledge of children. (Huijnen et al., 2017) participants perceived this same factor as critical to the robot's success, and also linked it to the potential for generalisability—especially in Wizard-of-Oz interfaces with direct and fine-grained adult control. A child could practice transfer even *within* robot interactions by working with different staff, or in different locations.

The “stepping stone” strategy (see also Vygotsky, 1978; or “social bridge” in Hughes-Roberts and Brown, 2015; Huijnen et al., 2016) assumes that children would successfully generalise skills from a robot interaction context to a human one, after sufficient practice. Yet, supporting autistic children to generalise, or transfer, their skills from the lab/intervention setting to a more real-world context is notoriously difficult (e.g., Schreibman et al., 2015). Concepts such as the “therapy register” (e.g., Johnston, 1988; Yoder et al., 2006) capture the issue of autistic children successfully learning and applying skills in one setting (e.g., speech and language therapy), but struggling to apply them in other relevant settings and situations (e.g., at home). Several studies that have specifically investigated autistic children generalising skills from technological contexts have not been particularly promising [e.g., see (Wainer and Ingersoll, 2011; Wass and Porayska-Pomsta, 2014; Whyte et al., 2015)]. With respect to technology-based autism tools, McCleery (2015) points out that there has been very limited, direct study of *near transfer* (i.e., skill transfer to another related task), and *far transfer* (i.e., skill transfer to other domains or naturalistic interaction contexts). The existing research has focused predominantly on screen-based technologies, over a wide range of ages and ability profiles, but not on social robots. More research is needed to test specifically whether robot-based activities can support near and far transfer of skills, and for *which* robots, activities, and subgroups of autistic learners (see section Conclusion). Following Huijnen et al. (2017), perhaps the role of adults in robot-based interventions, and in supporting successful transfer, should also be more overtly defined. For educators to see humanoid robots as potentially valuable and ethically acceptable tools, future research should focus on providing evidence of robots consistently supporting skill transfer into “real contexts.”

The interviewees' examples of potential future robot use also make a second critical assumption: that robots can actually teach autistic children new skills, particularly through implicit instruction. As with generalisation, this is not a settled question. Numerous social robotics studies have tested the *efficacy* of robots (i.e., whether a process can produce an intended result in a highly controlled setting), teaching autistic children specific, isolated skills such as point-following (e.g., David et al., 2018). Yet there are relatively few—if any—studies of robots' teaching *effectiveness* in non-lab contexts (though see Scassellati et al., 2018) and methodological issues mean many HRI studies do not provide clinically useful evidence (see Begum et al., 2016). Many of the skills that these educators wish to teach are also more complex than those in existing studies, with murkier criteria for success (e.g., a child understanding how her actions affect another person). Assuming that robots *could* facilitate skill transfer and show effectiveness in educational contexts, one outstanding question is whether robots could offer sufficient added value (vs. other technological/educational tools) to compensate for their current expense, fragility, and complexity.

### Personalisation, Content, and Teachers-as-Programmers

Strikingly, *none* of the educators made any reference to any kind of “robot app store,” or of otherwise buying or accessing pre-packaged curricula for robots, as they may already do with tablets or with some autism interventions. Instead, they repeatedly highlighted that successful robot use would need personalisation or adaptation of teacher-planned activities, especially given the enormous diversity of behaviours, preferences and traits of autistic learners. Directly or indirectly, respondents indicated that they (or people in teaching roles) should be the ones to implement whatever robot personalisation is required, with some explicitly explaining this in terms of programming (subtheme 3B). In both Hughes-Roberts and Brown (2015) and Huijnen et al. (2017), participants also stressed the need to personalise activities and robot behaviours (e.g., speech) to individual learners, suggesting that teachers would have responsibility over personalisation within the classroom, and even during the course of an interaction.

Yet, technical complexity and need for expertise were perceived as significant practical barriers to robot adoption. One participant in a leadership role described existing problems with teachers not using the personalisation capacities of existing technologies, such as apps, due to lack of training or time constraints. Others were concerned that technology expertise and training may be deliberately limited to single “experts,” and thus not easily “cascaded” through an entire teaching team. Other participants agree: Hughes-Roberts and Brown (2015) interviewees raised similar requirements for “the teacher [to] manipulate the robot without needing external support,” warning that “if it takes too long to set up the robot or deliver a lesson…[teachers] won't use (it)” (p. 52). Participants in Huijnen et al. (2016) cited as a particular strength of KASPAR that they would be able to use software to create interaction scenarios *themselves*, without specialist technical support. These views and concerns highlight a clear deployment challenge for robot developers and for educators: if the type of flexible robots that educators envision require extensive training or technical knowledge, they may struggle to gain traction in schools because of expertise bottlenecks, or overly complex, time-consuming procedures.

### What Type of Tools Are Robots? Educator Views vs. Current Research

The current findings suggest that autism educators at special schools in England have notably different expectations and priorities for humanoid robots than many existing HRI research projects, though share many points of agreement with other SEND and autism educators (Hughes-Roberts and Brown, 2015; Huijnen et al., 2017; though see Diep et al., 2015) and autism specialists working with other technologies (King et al., 2017). Educators expected that if they could access humanoid robots in the future, these would be *flexible tools for them and their teams*. They would be able to plan lessons using the same robot to work on different goals with individual learners or small groups, depending on need. This “flexible tool” view also agrees with a recent survey of UK-based teachers in regular, mainstream schools, where the second most popular proposed use of robots in schools was as a “versatile tool for the teacher, used in many situations” (Kennedy et al., 2016, Figure 6).

Yet, many existing autism-robotics and educational robotics research projects do not appear to be working toward a “flexible tools” endpoint. There are some clear practical reasons for that, including the difficulty of demonstrating feasibility and efficacy for a tool that could be used in almost any way, or investigating learning gains when every participant may have unique targets. Existing proof-of-concept and psychological experimentation work with robots (see section Introduction) often have basic science goals that add to the autism-robotics knowledge base and have focused on the needs of child users, rather than the needs of adult users who may operate robot systems. While the KASPAR research programme (e.g., Robins and Dautenhahn, 2017) has worked on iteratively developing and evaluating domain-specific robot-based lessons over time and has created customisation software for end-users to develop new learning scenarios, this capability does not appear to be well-known or well-documented compared to other aspects of the project (though see Huijnen et al., 2017). There are also several examples of packaged robot-based or robot delivered content. US-based Robokind manufactures humanoid robots, but has also developed and sells the “robots4autism” curriculum for autistic learners (https://robots4autism.com/). Scassellati et al. (2018) developed a month-long home-based social communication intervention for school-aged autistic children, using an autonomous robot. While both robots4autism and the Scassellati et al. (2018) system can present content adaptively to different children, neither offers the degree of flexibility and type of personalisation that educators within autism-specific special education settings seem to envision (e.g., programming the robot to use particular phrasing).

At present, the robotics industry may be offering something closer to educators' desired flexible use and to the “single, simple point of control” that Hughes-Roberts and Brown suggested (2015, p. 52). There are several tablet-based controls for commercially available robot NAO, such as the “AskNAO Tablet” app (Softbank and ERM Robotique https://www.asknao-tablet.com/), which offers a range of controls from push-button selection of pre-programmed actions to integrating with a powerful desktop program (Choreographe) for programming new robot behaviours. They also have a companion blocks-based visual programming language, AskNAO Blockly. Also using NAO, the EU-funded DREAM project developed a simplified, tablet-controlled version of their original autonomous system, DREAM Lite (Mazel and Matu, 2019), which therapists in Romania found fairly easy to learn and use, though they also requested further simplification (Cao et al., 2019). In addition to the contributions made by doing controlled robot experiments and developing specific teaching programmes, it would be a much-needed contribution for HRI and Human-Computer Interaction researchers and the commercial robotics industry to collaborate with educators, developing or modifying robot programming/control platforms to be both usable and secure.

## Limitations

This study is not without limitations. First, given the convenience sampling of participants, we cannot be sure that our findings reflect the views of autism educators in all special schools across England, or of educators working with autistic students in mainstream schools (in which the majority of autistic students are educated; Department for Education, 2018). Nevertheless, given the current interviewees' expertise in working with autistic students, particularly those with high support needs, they are likely to have provided particularly informed and nuanced views on the potential of robots as educational tools, as our findings attest.

A second key limitation is that the interviews prioritised the concerns of the larger DE-ENIGMA project in asking specifically about *humanoid* robots. Our respondents may have had different views and suggested other uses for animal-like robots such as Keepon (Kozima et al., 2007), or non-biomimetic robots. It is unclear whether the consensus present in the current dataset, such as using robots as “stepping stones” to human interaction, would also be present if discussing other robots. For the same reason, the interviews also specifically prompted respondents to consider applications for social and emotional skills teaching, but did not prompt them about academic or other applications, somewhat skewing the dataset in terms of the types of educational activities discussed.

## Conclusion

The findings of this study show multiple, strong points of agreements with how related participant groups (e.g., Hughes-Roberts and Brown, 2015; Huijnen et al., 2016, 2017, 2019) have conceptualised robots as potential tools for autism education. Importantly, our educators were not uncritically approving of the use of robots in the classroom (see also (Serholt et al., 2017), for similar views from mainstream educators). Rather, they carefully outlined specific use-cases and circumstances in which robots were predicted to be beneficial (e.g., as “stepping stones” to social interaction), and conditions that would need to be met to ensure their adoption in the classroom, including integration with educational curricula, and the capacity to personalise robots to meet the specific needs of individual, autistic learners.

The findings suggest several promising avenues for future research. First, educators repeatedly highlighted the idea, prevalent in HRI literature, that robots' predictability and consistency of behaviour should benefit autistic learners in particular (e.g., Rudovic et al., 2017; Straten et al., 2018); it should reduce demands on them, put them at ease, and potentially facilitate learning. These claims are logical based on the diagnostic features of autism and current educational practices that aim to offer children predictability and structure at school (e.g., Mesibov and Shea, 2010), as well as theories of autistic perception and information processing (e.g., Pellicano and Burr, 2012; Lawson et al., 2014). However, they have not been rigorously operationalised and evaluated at a behavioural level. Research is required to test these widely-held beliefs about the benefits of robot predictability and exactly how it may affect children in learning contexts.

Second, the capacity of humanoid robots to support autistic children in developing transferrable, generalisable skills is not currently supported by clear research evidence. Given the centrality of educator views that robots need to be a stepping stone to human-human interaction, investigating skill transfer should be an urgent priority. Further generalisation studies might also test educators' beliefs, as expressed herein, that a humanoid robot might *better* teach, or support transfer of, social skills, than would other robot morphologies. These questions are not only the domain of autism education researchers; they should also concern robotics researchers. Based on this current research, robots for autism education—no matter how appealing or user-friendly—would not meet educators' and children's needs if they did not consistently support skill transfer. Robots that only facilitate learning gains within robot-based activities (i.e., *training effects*) are unlikely to be ethically or financially justifiable for educators or the broader autism community.

Educator interview studies are a valuable source about of information for robotics researchers and industry about the needs of child and adult users, but are not in themselves sufficient to bridge the “deployment gap” between preliminary, lab-based research, and the vision of robots as educational tools. Huijnen et al. describe this gap perfectly, writing:

“For socially interactive robots to actually make a difference to the lives of children with ASD and their carers, they have to find their way out from case studies with ‘standalone' robots in robotics labs to… education environments as part of daily activities/therapies. Being effective in eliciting a certain target behaviour of a particular child in a lab environment, will not automatically ensure… adoption of use by professionals in the field” (2016, p. 446).

Greater engagement with educators—and other key stakeholders, including autistic children themselves—during design, implementation, and evaluation should help to ensure that the resulting robotics systems and programmes are relevant to autistic learners and those who support them, sufficiently tailored to the realities of their everyday learning contexts, and consistent with their values (e.g., Lloyd and White, 2011). Such participatory processes are being championed across autism research (Nicolaidis et al., 2011; Pellicano and Stears, 2011; Fletcher-Watson et al., 2019), but especially within technology-related autism research (Frauenberger et al., 2011; Porayska-Pomsta et al., 2012; Brosnan et al., 2016). The children's interaction design community can offer useful examples and methodological guidance for undertaking participatory technology research with educators and children, including children on the autism spectrum (e.g., Frauenberger et al., 2013).

In advocating for HRI researchers to engage more fully with autism education practitioners while planning, developing, and evaluating robotic tools, we realise that this could pose a substantial change to many established ways of working, and that fully co-produced research might not be possible on many projects. Yet stakeholder participation in research—beyond being a passive participant or subject—can take many forms, including as advisors, as consultants, or as full decision-making partners throughout a project. The risks of designing robots that do not consider stakeholders' views, needs and contexts could be far-reaching for research and industry, especially given the costs of developing and deploying robots.

The current findings highlight that there will be no one-size-fits all design “solution” for robotics in autism education, and that current “solutions” may pose later challenges for autistic children. Such future work therefore needs to involve key stakeholders in the design and implementation process (see also Serholt et al., 2017), designing *with* educators, parents and autistic children, rather than *to, on*, or *for* them, to ensure that this work has a direct and sustained impact on those who need it most. This process will require beginning from a point of rigorously co-investigating the assumed and predicted benefits of robotics for autistic children, and balancing these against potential interpersonal, developmental, and resource costs. We envision that robot design driven by technical innovation will be increasingly combined with—or shaped by—approaches that prioritise the needs and values of users.

## Data Availability Statement

The datasets generated for this study will not be made publicly available because participants did not consent to future re-use of their interview data by other researchers.

## Ethics Statement

The studies involving human participants were reviewed and approved by The UCL Institute of Education Research Ethics Committee (REC857). The patients/participants provided their written informed consent to participate in this study.

## Author Contributions

VC, SP, BS, TT, and EP devised and piloted the interview schedule. EA, SM, and AA recruited participants. EA, SM, and AA interviewed participants. AA and EP analysed the data. AA and EP drafted the manuscript. All authors commented on and edited the manuscript prior to submission.

### Conflict of Interest

The authors declare that the research was conducted in the absence of any commercial or financial relationships that could be construed as a potential conflict of interest.

## Footnotes

^1^We use “identify-first” language (“autistic person”) rather than person-first language (“person with autism”), because it is the preferred term of autistic activists (e.g., Sinclair, 1999) and many autistic people and their families (Kenny et al., 2016) and is less associated with stigma (Gernsbacher, 2017).
