# Supplementary material for: Educators' Views on Using Humanoid Robots With Autistic Learners in Special Education Settings in England
Source: Front Robot AI. 2019 Nov 1;6:107. doi: 10.3389/frobt.2019.00107 (PMC7805648; doi:10.3389/frobt.2019.00107)
Supplement: Supplementary file 1 [file Table_1.docx]

Supplementary Material

**Supplementary Table 1.**

Participant background information.

|  | **Gender** | **Current role** | **Experience in current school (in years)** | **Age, needs and language of the autistic children with whom the participant works** | **Type of school** |
| --- | --- | --- | --- | --- | --- |
| Interview | Female | Primary teacher | 1 year | Students aged 10 (year 5), varied levels of spoken language | SEN school, which includes autistic children |
| Interview | Female | Music teacher | 10 years | Autistic children, young adults with a variety of abilities (specialises in SEND) | Multiple SEN settings |
| Interview | Female | Teaching assistant | 0.5 years | Early Years (4-5 year-olds). Students “high and low” ability | Specialist school for autistic pupils |
| Interview | Female | School founder and Headteacher | 10 years | School Headteacher of 6-11 year-olds | SEN school, which includes autistic children |
| Interview | Female | Primary teacher | 4 years | Year 3 autistic students (8-year-olds), with additional need and varying spoken language abilities | SEN school, which includes autistic children |
| Interview | Female | Secondary music teacher | 4.5 years | Reception to Year 13 (4-19 year-olds) and students with various needs, speaking and non-speaking, mild to severe learning difficulties, complex needs | SEN school, which includes autistic children |
| Interview | Female | Primary teacher | 2.5 years | Year 3 students (8-year-olds) with varying spoken language abilities. Works mainly with autistic students | SEN school, which includes autistic children |
| Interview | Female | Secondary humanities teacher | 5 years | 11-16 year-old, “severely autistic” students with varying spoken language abilities, ranging from speaking to non-speaking | SEN school, which includes autistic children |
| Interview | Male | Primary teacher | 1 year | Year 4 (8-9 years), various abilities, speaking and non-speaking | SEN school, which includes autistic children |
| Interview | Male | Primary cover teacher | 7 years | Reception to Year 6 students (4-11 year-old) autistic students and/or with global developmental delay | SEN school, which includes autistic children |
| Interview | Female | Secondary ICT teacher | 3 years | Sixth form aged students (16-19 year-olds) focused on Work Experience & Travel Training | SEN school, which includes autistic children |
| Interview | Female | Primary teacher | 10 years | Year 1 (6-year-old) autistic children both speaking and non-speaking | SEN school, which includes autistic children |
| Interview | Female | Science secondary teacher | 3 years | Autistic children with and without moderate intellectual disability, both speaking and non-speaking | SEN school, which includes autistic children |
| Interview | Male | Primary teacher | 5 years | Year 6 (10-year-old) students, both speaking and non-speaking | SEN school, which includes autistic children |
| Focus group 1 n=5 | Male | KS2 Primary Teacher | 0.25 years | Years 3-6 (7-11 year-olds), mixed abilities regarding spoken language and use of supports | Autism resource unit, attached to a mainstream school |
|  | Female | Early Years Teacher | 3 years | Nursery to year 1 autistic students (3-6 year-olds), speaking and non-speaking | Autism resource unit, attached to a mainstream school |
|  | Female | Leader of autism unit & KS1 classroom teacher | 2.5 years | Years 1-2 (5-7 year-old) students, varied abilities, including children who are speaking and non-speaking | Autism resource unit, attached to a mainstream school |
|  | Female | Inclusion teacher | 2 years | Students with various needs and abilities, including learning disabilities and sensory processing needs | Autism resource unit, attached to a mainstream school |
|  | Female | Speech and language therapist | ~~-~~ | Autistic students with varied abilities | Autism resource unit, attached to a mainstream school |
| Focus group 2 n=6 | Female | Teaching assistant | 1.5 years | Autistic children aged 7-9 years. Most able classes in primary, 1 non-speaking child | SEN school, which includes autistic children |
|  | Female | Teacher | 6 years | Year 5 (9-11 year-old) students, four of whom are autistic, with mixed abilities and learning needs | SEN school, which includes autistic children |
|  | Female | Assistant head of primary | 12 years | Not teaching this year, works with whole school | SEN school, which includes autistic children |
|  | Female | Secondary teacher | 4 years | Sixth form teacher (16-19yr olds) of three autistic students; all speaking, although two use AAC device | SEN school, which includes autistic children |
|  | Female | Speech and language therapist | 1 year | Works with primary and secondary students, range of spoken language abilities | SEN school, which includes autistic children |
|  | Female | Occupational therapist | 1 year | Works with any child who is referred in the school from primary and younger, mixture of ages and abilities | SEN school, which includes autistic children |
| Focus group 3 n=6 | Female | Lead teaching assistant | 18 years | Early Years and Years 1-2 (4-7 year-olds). Children with varying needs, many non-speaking students; uses PECS. | Specialist school for autistic pupils |
|  | Female | Primary teacher | 7 years | 4-5 year-olds, some speaking and some non-speaking | Specialist school for autistic pupils |
|  | Female | Occupational therapist | - | Years 1-2 (5-7 year-olds) and Years 3-6 (7-11 year-olds), mixed needs abilities, many non-speaking students | Specialist school for autistic pupils |
|  | Male | Teacher training, technology specialist | 7 years | Delivers across the unit, and works with Years 5-6 (10-11 year-olds) in primary school | Specialist school for autistic pupils and unit |
|  | Male | Head teacher | 7 years | Not teaching. Works across school for autistic children with mixed abilities and needs, many non-speaking | Specialist school for autistic pupils |
|  | Female | Speech and language therapist | 0.5 years | Early years and Years 1-2 (4-7 year-olds) and secondary | Specialist school for autistic pupils |

Notes: AAC = Augmentative and Alternative Communication; Headteacher = school principal; KS1 = Key Stage 1 (Years 1-2; 5-7 year-olds); KS2 = Key Stage 2 (Years 3-6, 8-11 year-olds); PECS = Picture Exchange Communication System (Bondy & Frost, 1994); SEN = Special Educational Needs; SEND: Special Educational Needs and Disabilities; Sixth form = 16-19 year-olds; TA = teaching assistant.
