# Supplementary material for: Educators' Views on Using Humanoid Robots With Autistic Learners in Special Education Settings in England
Source: Front Robot AI. 2019 Nov 1;6:107. doi: 10.3389/frobt.2019.00107 (PMC7805648; doi:10.3389/frobt.2019.00107)
Supplement: Supplementary file 2 [file Table_2.docx]

Supplementary Material

Supplementary Table 2.

Full interview schedule used in the current studies, with questions in order of discussion

| **Tell me about the overall hopes and aspirations that you have for your pupils on the autism spectrum?**  Follow-up probes  How do you communicate these hopes and aspirations to staff and parents?  *How do you determine whether these aspirations are appropriate?* |
| --- |
| **Give me, briefly, a broad description of the curriculum you have in place in your setting?**  Follow-up probes  To what extent is this based on a ‘national curriculum, similar to what is delivered to neurotypical children?  Tell me about the ways in which you modify your curriculum for individuals and groups  In what ways (if any) are other professionals involved in the education of pupils at your school?  Tell me about how you measure the progress of your autistic pupils (e.g., academic process, social progress, challenges in doing so). |
| **Tell me about the specific programmes used in your school**  **(e.g., Picture Exchange Communication System (PECS), Treatment and Education of Autistic and related Communication handicapped Children (TEACCH), Applied Behaviour Analysis (ABA), Sensory integration, etc.)**  Follow-up probes  Are they used with specific pupils or all pupils?  What do you feel is the main aim of these programmes?  How do you review and evaluate whether these programmes ‘work’ with your pupils, and achieve their aims?  Tell me about how you ensure that your staff members are kept up-to-date on knowledge about autism and, particularly, educating pupils on the autism spectrum. |
| **Can you tell me about the strategies you use to teach social and emotional skills in your setting?**  Follow-up probes  Could you tell us some examples of social and emotional skills on which you usually focus for your pupils, which are difficult to teach?  Could you tell us some examples of specific activities you use for teaching socio-emotional skills?  How difficult or easy is it to teach socio-emotional skills with the current tools/methods? Are there specific skills that are easier or harder to teach? Are there specific parts of the skill that are difficult to teach, such as generalisation to different contexts?  More specifically, what kind of practices, games, technologies do you use to teach your pupils socio-emotional skills? |
| **What kind of tools or props do you use? Do you use any sort of technology (e.g., iPad/tablet, sensory floors etc.)? What are the advantages and disadvantages of using such tools and how do the children react to them?**  Follow-up probes  Do you think you could teach social and emotional skills to children in a better way if you had some tools or piece of technology available to you? If so, how?  Do you use any types of technology as part of the specific programmes? |
| **Now, let’s suppose that one of the tools that you can use is a human-like robot – a robot that has human-like features, such as arms, hands, legs, a head and is able to be programmed to talk.**  **How do you feel about the use of robots in schools for pupils on the autism spectrum?**  Follow-up probes  Can you mention ways in which a human-like robot could be integrated to activities for children’s learning? In the context of these activities, what kind of goals could you address with the use of a human-like robot?  In what ways do you think a human-like robot could be used to help your autistic pupils?  Might a human-like robot be useful for helping with children’s social and emotional learning?  How you do think your pupils would respond to a human-like robot? Would you need to make any adjustments to ensure that children could access the robots in teaching  Do you have any concerns about the use of robots? |
